# An Integrated Insight into the Relationship between Soil Microbial Community and Tobacco Bacterial Wilt Disease

**DOI:** 10.3389/fmicb.2017.02179

**Published:** 2017-11-07

**Authors:** Hongwu Yang, Juan Li, Yunhua Xiao, Yabing Gu, Hongwei Liu, Yili Liang, Xueduan Liu, Jin Hu, Delong Meng, Huaqun Yin

**Affiliations:** ^1^College of Agronomy, Hunan Agricultural University, Changsha, China; ^2^School of Minerals Processing and Bioengineering, Central South University, Changsha, China; ^3^Key laboratory of Biometallurgy, Ministry of Education, Changsha, China

**Keywords:** tobacco wilt disease, plant health, soil microbial community, microbial community composition, molecular ecology networks

## Abstract

The soil microbial communities play an important role in plant health, however, the relationship between the below-ground microbiome and above-ground plant health remains unclear. To reveal such a relationship, we analyzed soil microbial communities through sequencing of 16S rRNA gene amplicons from 15 different tobacco fields with different levels of wilt disease in the central south part of China. We found that plant health was related to the soil microbial diversity as plants may benefit from the diverse microbial communities. Also, those 15 fields were grouped into ‘healthy’ and ‘infected’ samples based upon soil microbial community composition analyses such as unweighted paired-group method with arithmetic means (UPGMA) and principle component analysis, and furthermore, molecular ecological network analysis indicated that some potential plant-beneficial microbial groups, e.g., *Bacillus* and *Actinobacteria* could act as network key taxa, thus reducing the chance of plant soil-borne pathogen invasion. In addition, we propose that a more complex soil ecology network may help suppress tobacco wilt, which was also consistent with highly diversity and composition with plant-beneficial microbial groups. This study provides new insights into our understanding the relationship between the soil microbiome and plant health.

## Introduction

Soil borne pathogens attack crops and causing huge yield losses. Bacterial wilt disease, caused by *Ralstonia*, can infect *Solanaceae* crops (e.g., tobacco, tomato, egg plants, etc.) with large scale crop damage world wide, and many efforts have been made to control this disease (Janvier et al., 2007). A number of factors have an effect on soil borne plant pathogen infections including the soil microbiome (Pieterse and Dicke, 2007; Delgado-Baquerizo et al., 2016). The importance of soil microbes in controlling of plant disease is well recognized and soil microbiome links to plant disease in many ways: stimulating production of plant growth hormones, competing with pathogens for nutrients, production of compounds (e.g., antibiotics) that inhibit pathogens or induce plant resistance to pathogens (Berendsen et al., 2012; Mendes et al., 2013). Because controlling plant pathogens by microbes is a sustainable and chemical-free approach, disease-suppressive soils with beneficial microbes have been developed for biocontrol of plant diseases (Weller et al., 2002; Penton et al., 2014; Cha et al., 2016). The incidence or severity of disease is often lower in suppressive soils in comparison with that in surrounding soils (Cook and Baker, 1983).

Concerns about the impact of the soil microbiome on plant health has been examined in several studies (Sanguin et al., 2009; Qiu et al., 2012; Penton et al., 2014; Santhanam et al., 2015; Cha et al., 2016; Latz et al., 2016; Niu et al., 2016, 2017; Wang et al., 2017). Soil microbial community diversity, composition, function, and ecological relationship are all associated with plant soil borne disease outbreaks. Using a high-through put sequencing approach, Wang et al. (2017) found the microbial community was different between healthy and bacterial wilt-infected fields. Niu et al. (2016) also found that cropping regimes affected plant disease outbreaks by changing the soil microbial communities. Addition of organic fertilizers could inhibits plant diseases through alteration of soil microbial composition, diversity, and activity (Hunter et al., 2006). However, most studies have focused on the diversity and composition of the soil microbiome but ignored the microbial interactions.

Microbial interactions are a vital part of the soil microbiome (Zhang et al., 2014) and the interactions among different microbial groups are important for determining the ecosystem functioning/stability (Zhou et al., 2011), thus maybe a more integrated, complex network (many, stable interactions) is more difficult for a pathogen or other intruder to enter. Therefore, we hypothesized that complex network played some role in suppression of tobacco bacterial wilt disease. Many network approaches have been developed to reveal the interactions among microbial groups, e.g., equation-based network (Yeung et al., 2002), Bayesian network (Gerstung et al., 2009), relevance network (Horvath and Dong, 2008), CoNet network (Detti et al., 2011; Soffer et al., 2015) and random matrix theory (RMT)-based network (Deng et al., 2012). The threshold selection in the network construction is important, because the choice of thresholds have important effects on which OTUs are selected (Poudel et al., 2016). A RMT-based network can automatically identify the threshold for network construction (Zhou et al., 2010) using 16S rRNA gene sequencing data creating a molecular ecology network that is objective rather than subjective (Zhou et al., 2011; Deng et al., 2012).

The aim of the present study is to evaluate the relationship between soil microbiome and plant health and to reveal which microbes may play a role in inhibiting bacterial wilt disease. To reveal this relationship, we sampled soil samples in 15 tobacco farmlands (8–10 samples in each farmland) that were located in three different regions in central south of China. We investigated the soil microbial community using 16S rRNA gene sequencing, constructed molecular ecological networks based on random matrix theory, analyzed network properties and inferred the key microorganisms in these networks. As a result, the present study offers an integrate insight into the relationship between soil microbial community and tobacco wilt disease.

## Materials and Methods

### Soil Samples and Wilt Infection Rate

Soil samples were collected from 15 different tobacco fields located in different regions of Hunan province. Detailed location and other information are list in Supplementary Table S1. Eight to 10 samples were taken from each filed. Soil samples were collected using checkerboard sampling method as described in Niu et al. (2017). To be specific, each tobacco field was divided into 10 areas and upper layer soils (0–20 cm) at the central point of each area were sampled as one sample. The samples were collected on 28th July, 2015. Soil samples were stored at -80°C before DNA extraction.

The wilt infection rate was calculated by the percentage of infected plants in each field. That is,

$$\begin{array}{c} \mathrm{Infection}\;\mathrm{rate}\;=\;Ni/Nt\;\times \;100\%, \end{array}$$

where Ni and Nt represents the number infected plants and the total number of plants in each field (about 250 plants), respectively.

### DNA Extraction, PCR Amplification, Sequencing and Data Processing

DNA extraction, amplification of 16S rRNA amplicons and sequencing were performed as described in our previous studies (Niu et al., 2016; Niu et al., 2017). Briefly, DNA were extracted using a MO BIO PowerSoil DNA Isolation Kit (MO BIO, San Diego) following the manufactures’ manual. PCR amplification of 16S rRNA V4 region was performed on a Biosystems 2720 Thermal Cycler (ABI Inc., United States) with the primer pair 515F (5′-GTGCCAGCMGCCGCGGTAA-3′) and 806R (5′-GGACTACHVGGGTWTCTAAT-3′) primers together with Illumina adapter sequences and barcodes. The PCR reactions consisted of 0.2 μl primers (10 nM each), 12.5 μl Taq Master Mix (Vazyme, Piscataway, NJ, United States), 1.0 μl DNA template and ddH2O to a final volume of 25 μl. PCR amplifications were performed as follows: desaturated at 95°C for 5 min; followed by 30 cycles of 30 s at 95°C, 30 s at 55/59/62°C and 30 s at 72°C and a final extension step at 72°C for 10 min. PCR products were recovered using gel extraction kit (OMEGA Bio-Tek Inc., Doraville, GA, United States) after subjecting the PCR mixture to agarose gel. Quality and quantity of recovered products were measured on Nanodrop Spectrophotometer (ND-1000 Spectrophotometer, Nanodrop products, Wilmington, DE, United States). Sequencing libraries were constructed using 200 ng of each purified PCR product and sequencing was performed on Illumina MiSeq machine (Illumina, San Diego, CA, United States) using the MiSeq 500 cycles kits. The Raw data were in *fastq* format. The 16S rRNA gene sequences have been submitted to NCBI SRA database following the accession number of KR831285 – KR 855564.

Processing of sequencing data were performed on an in-house galaxy pipeline developed in the lab of Dr. Zhou^1^. Firstly, reads were assigned to different samples according to their barcode sequences and primer sequences were then removed. Subsequently, low quality reads (QC threshold < 20) were removed using Btrim (Kong, 2014). Then, the forward and reverse reads with at least 10 bp overlap and less than 5% mismatches were merged using Flash (Magoc and Salzberg, 2011), ambiguous bases (N) were removed from the merged sequences. Finally, reads with 97% similarity (Clustering threshold) were assigned to the same OTU, chimeras and singletons were removed and OTU table was generated using UPARSE (Edgar, 2013). To eliminate the influences caused by different sequencing depth, samples were normalized to 19, 000 for further analysis. All downstream analyses including microbial community analyses and molecular ecology network construction were carried out using the normalized OTU table. Taxonomic assignment was carried out by blasting the sequences to RDP (Ribosomal Database Project) database with a 50% minimal confidence.

### Random Matrix Theory Based Molecular Ecology Networks

Molecular ecology networks (MENs) were constructed based on OTU abundance in each tobacco field, yielding a total of 15 networks. Only OTUs presented in 9 out of 10 (For site D, 8 out of 9 and for J, 7 out of 8) replicate samples were used for network construction. Because the criteria for selecting OTUs in network construction was different for site D and J, further analyses were carried out on the other 13 networks, and the results were similar to that obtained from 15 networks (data not shown). Networks were constructed as follows: paired valid missing values (a relative abundance of 0) was replaced by 0.01, logarithm normalization of relative abundance was carried out to reduce the microbial community compositional bias (Faust and Raes, 2012), and similarity matrix was constructed based on Pearson Correlation Coefficient. Random matrix theory was used to choose the similarity threshold (*St*) automatically (Zhou et al., 2010, 2011; Deng et al., 2012). For network analysis, we characterized the global network properties and used greedy modularity optimization for module separation. All network analyses were performed on Institute for Environmental Genomics, The University of Oklahoma website^2^. Networks were visualized using Gephi software. Modules in each network were randomly colored, and modules with less than five nodes were colored gray.

### Statistical Analyses

All calculations and statistical analyses were performed on R statistical platform (v 3.4.0). Community analyses were carried out based on OTU relative abundance in each tobacco planting field. Diversity indexes including Shannon diversity index, evenness and Chao1 index were calculated using the ‘vegan’ package. Principle component analysis (PCA) was carried out using the ‘vegan’ package. Analysis of similarity (ANOSIM) and unweighted paired-group method with arithmetic means (UPGMA) based on Bray–Curtis distance matrix. To investigate any differences in microbial community in sites with different wilt infection rate, PCA, UPGMA, and ANOSIM were performed using the average relative abundance of 8–10 replicates in each site. Partial Least Squares Path Modeling (PLSPM) was performed using ‘amap,’ ‘shape,’ ‘diagram,’ and ‘plspm’ package. Pearson correlation analysis was used to calculate the correlation between two parameters. Significance level of difference between different groups was determined by one-way ANOVA (analysis of variance) followed by the Tukey’s test. For unbalanced design, one-way ANOVA followed by Tukey’s test was carried out using the General Linear Model function in Minitab 16.0 (Minitab inc.). A *p*-value of less than 0.05 was considered as significant.

## Results

### Soil Properties and Wilting Symptom

The bacterial wilt infection rate ranged from 1.19 to 80.45% in all 15 sites (Supplementary Table S1). Six sites had an infection rate of less than 25%, five sites had infection rates between 25 and 50% and four sites had an infection rate more than 50%. Nine sites were the continual cropping of tobacco (Continues), while six sites were the rotation cropping of tobacco and other crops including lily, turnip and maize (Rotation). There is no statistically significant difference (*p* > 0.05, ANOVA following Tukey’s test) of infection rate among sample locations, nor any significant difference (*p* > 0.05, student *T*-test) between cultivation regimes (Rotation and Continual cropping).

Soil properties including pH, water content, Potassium, Calcium and Ion contents were shown in Supplementary Table S2. Among them, pH (Pearson = -0.606, *p* = 0.017) and Calcium (Pearson = -0.591, *p* = 0.020) showed significantly negative correlation with wilt infection rate (Supplementary Figure S1), and the remaining three parameters didn’t show significant correlation with plant wilt symptom.

### Microbial Community

**FIGURE 1:**
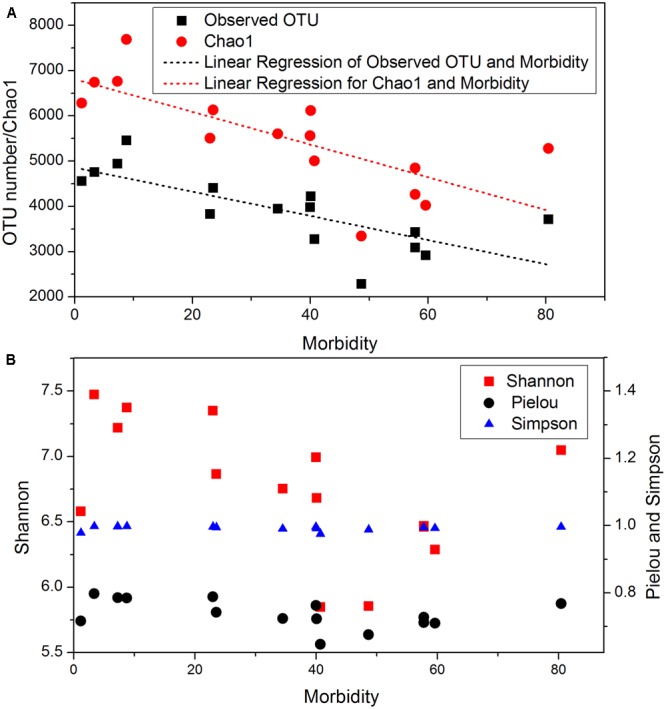
Microbial community diversity in 15 sites with different bacterial wilt infection rate. **(A)** Observed OTU and Chao1; **(B)** Shannon, Pielou and Simpson indexes. Linear regressions were present for indexes that significantly (*p*-value < 0.05) correlated with infection rate (Morbidity). Pearson correlation indexes and *p*-values are shown in Supplementary Table S5.

A total of 8,366,296 reads were obtained from 147 samples, and 21,429 OTUs were identified from the reads. The rarefaction curves (Supplementary Figure S2) indicated that the sequencing depths were sufficient for downstream analysis. Fifteen sites had significantly different OTU numbers and Chao1 indexes. Pearson correlation showed the mean observed OTU number and Chao1 index showed significant (*p* < 0.05) negative correlation with bacterial wilt infection rate (**Figure 1A**). In addition, all sites had similar Simpson diversity index (**Figure 1B**, from 0.975 to 0.995) and Pielou evenness (0.65–0.79). The Shannon diversity index differed among sites, and weakly correlated with wilt infection rate (Pearson index = -0.485, *p* = 0.067).

**FIGURE 2:**
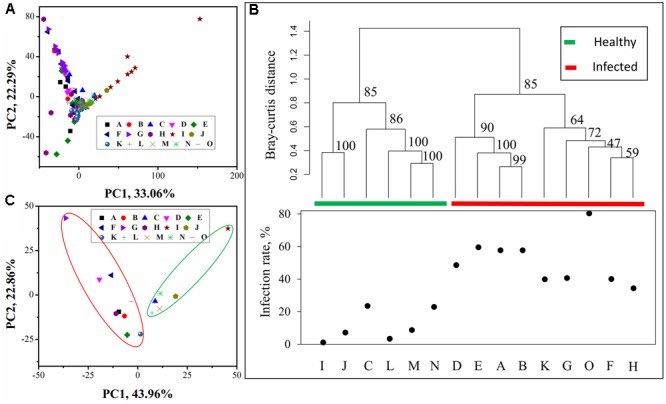
Principle component analysis (PCA) and Unweighted paired group method with arithmetic mean (UPGMA) tree of microbial community composition. **(A)** PCA plot of all 147 samples grouped by sample sites; **(B)** UPGMA tree constructed from ‘Bray–Curtis’ distances between microbial community in each site, sites were clustered into two groups based on the community composition and as the two groups differed in the wilt infection rate, they were referred to ‘healthy’ (which had the infection rate lower than 25%) and ‘infected’ group (which had the infection rate higher than 25%), respectively. The numbers associated with the branches refer to bootstrap values (confidence limits) resulting from 100 replicate resamplings; **(C)** PCA plot of microbial community in sample sites. In **(B,C)**, average relative abundance in each site was used for analysis.

Principle component analysis plot of all 147 samples (**Figure 2A**) showed that samples from individual sites grouped well with each other. The analysis of similarity (ANOSIM) (*R* = 0.814, *p* = 0.001) indicated that there were significant differences between groups (sites). To investigate the relationship between soil bacterial community composition and tobacco wilt disease, UPGMA tree (**Figure 2B**) and PCA (**Figure 2C**) were performed based on the average OTU relative abundance. UPGMA tree showed that samples with similar wilt infection rates often clustered together. The two branches differed obviously in their wilt infection rate. The left branch was composed of five sites (I, J, C, L, M, and N) whose wilt infect rate was lower than 25% and we referred to these sites as ‘healthy.’ The ‘infected’ sites (D, E, A, B, K, G, O, F, and H) are on the right branch. In addition, the ‘infected’ branch was further separated into the ‘severely infected’ [>50%, with site D (48.67%) as the exception] and the ‘moderately infected’ (25–50%, with the site O (80.48%) as the exception) branch. PCA plot of the bacterial communities in 15 sites showed similar results as UPGMA tree, ‘healthy’ and ‘infected’ groups separated along the first PCA axis. The ‘healthy’ sites are in the green circle, while the ‘infected’ sites are in the red circle. When all samples were classified into two groups (two branches on the UPMGA tree) according to the wilt infection rate, similarity analysis (ANOSIM) showed that the distances between groups was significantly larger than that within the groups (*R* = 0.7771 and *p* = 0.001), suggesting that the two groups differed significantly in their microbial community composition.

**FIGURE 3:**
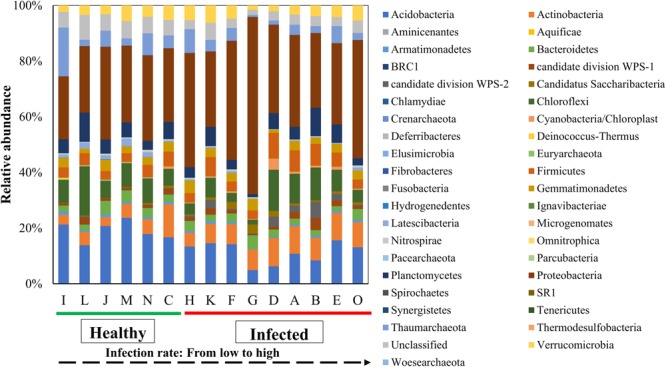
Average relative abundance of bacterial phylum in soil samples. Results are means of 10 replicates. All sites can be grouped into two groups: ‘Healthy’ that has the infection rate of less than 25% and ‘Infected’ that has the infection rate more than 25%.

All OTUs were classified into 931 genera and 39 phyla. The most abundant phylum in soils was the *Proteobacteria* which account for 33.49% of the bacterial community (**Figure 3**). Two phyla, *Actinobacteria* and *candidate division WPS-2* significantly positive correlation with wilt infection rate (Supplementary Table S3). Ten phyla showed significantly negative correlation with wilt infection rate, which included *Acidobacteria* (Pearson = -0.629, *p* = 0.012), *BRC1* (Pearson = -0.762, *p* = 0.001), *Crenarchaeota* (Pearson = -0.573, *p* = 0.026, *Euryarchaeota* (Pearson = -0.612, *p* = 0.015) *Hydrogene*-*dentes* (Pearson = -0.559, *p* = 0.030), *Ignavibacteriae* (Pearson = -0.607, *p* = 0.016), *Latescibacteria* (Pearson = -0.651, *p* = 0.009), *Pacearchaeota* (Pearson = -0.737, *p* = 0.002), *Synergistetes* (Pearson = -0.677, *p* = 0.006), and *Woesearchaeota* (Pearson = -0.546, *p* = 0.035). The most abundant genus in soils was Acidobacteria_GP16 (5.31%), followed by *Nitrososphaera* (4.97%), *Sphingomonas* (3.66%), and *Gemmatimonas* (3.06%), and the genus *Acidobacteria_GP16* showed significantly negative correlation with tobacco wilt infection rate (Pearson = -0.641, *p* = 0.010). As we focused on the wilt disease caused by *Ralstonia*, we investigated the relationship between relative abundance of the genus *Ralstonia* and the wilt disease. The genus *Ralstonia* had relative low abundance in soils (0.002–0.21%) and showed significantly positive correlation with tobacco wilt morbidity (Pearson = 0.518, *p* = 0.048).

### Molecular Ecology Networks

**FIGURE 4:**
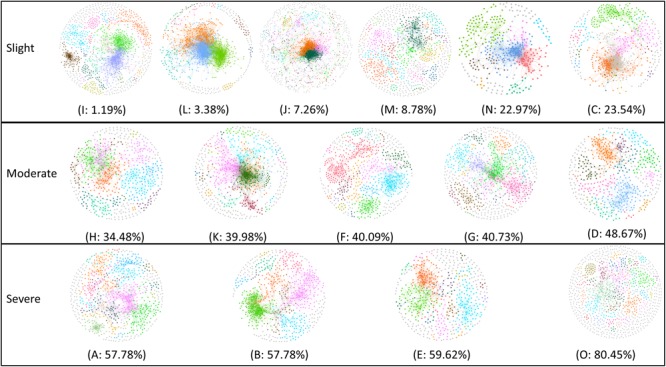
Random matrix theory (RMT)-based molecular ecology networks in soils during Tobacco cultivation. Networks are present following the order of Tobacco bacterial wilt infection rate (from low to high). In the networks, nodes represent OTU and links represent significant correlation, modules are randomly colored and modules with less than five nodes are colored gray. Sites with relative low infection rate (<25%, including I, L, J, M, N, and C) were referred to ‘healthy’ group, sites with relative high infection rate (>50%, including H, A, B, E) were referred to ‘severe’ group and others that has the infection rate between 25 and 50% (K, F, G, O and D) were referred to ‘moderate’ group.

To unravel the relationship between soil microbe–microbe interactions and tobacco bacterial wilt disease, we constructed a co-occurrence network for each field (**Figure 4**). The automatically generated similarity threshold (*st*) of the networks ranged from 0.89 to 0.94 (Supplementary Table S4) and power-low *R* square ranged from 0.82 to 0.93 (Supplementary Table S4). The *st* were chosen using the RMT-based theory to obtain an adjacency matrix, which represents the strength of the connection between each pair of OTUs. Similar *St* indicated that the minimum strength of the connection in all networks was similar. The *R* square values were near to 1 indicating that the goodness of fit was proper. The network properties differed obviously among sites (Supplementary Table S4), for example, the nodes ranged from 398 to 1436, the links ranged from 1139 to 12580, the average degree ranged from 2.35 to 17.90 and the modules ranged from 30 to 208. The sites that with healthy plants tended to have more complex networks based on the average degree and number of links that showed significant negative correlation with wilt incidence. The network complexity can be described by the number of links and average connectivity (average *K*) with more links and higher average *K* indicative of a more complex network (Deng et al., 2012). Furthermore, modularity is an important concept in networks and microbial populations in the same module have similar ecological niches (Deng et al., 2012). Pearson correlation analysis indicated that average *K* (Pearson = -0.656, *p* = 0.008), number of links (Pearson = -0.684, *p* = 0.005) and number of modules (Pearson = -0.543, *p* = 0.037) showed a strong negative correlation with Tobacco bacterial wilt infection (**Table 1**), meaning sites with more complex and more modular networks have lower wilt infection rate.

**Table 1:** Pearson correlation between wilt infection rate and microbial community which are related to microbial community diversity, composition and network properties.

		Diversity				Composition		Network			
		Diversity	Evenness	Observed OTU	Chao1	PC1	PC2	Nodes	Links	Avg.K	Modules
Wilt	Pearson	–0.485	–0.402	–0.751	–0.747	–0.639	–0.252	–0.317	–0.684	–0.656	–0.543
	*p*-value	0.067	0.137	0.001	0.001	0.01	0.365	0.250	0.005	0.008	0.037

*Avg.K, average degree.*

### Network Key Taxa

To reveal the key microbes in the networks, we looked into the nodes with max degree, max betweenness, max stress centrality, and max eigenvector centrality (**Table 2**). Nodes with max degree are the nodes that have the most interactions with other nodes (Chaffron et al., 2010; Deng et al., 2012). Betweenness and stress centrality are similar to each other, nodes with highest betweenness (or stress centrality) can serve as brokers (Guimera et al., 2007; Deng et al., 2012). The highest eigenvector centrality means the nodes have the highest degree when they connected to other central nodes (Bonacich, 1987). These nodes are important in maintaining the microbe-microbe interactions in the networks. **Table 2** shows the key OTUs and their taxa in all networks. Members in phylum *Proteobacteria* and *Acidobacteria* are the main key taxa ‘healthy’ networks (e.g., members in phylum *Acidobacteria* acted as key taxa in I, J, and C, while members in phylum Proteobacteria in I, M, N, and C). Members in phylum *Actinobacteria* (e.g., genus *Gaiella*) and *Firmicutes* (e.g., genus *Bacillus*) are also important taxa in networks from fields with healthy plants. Key taxa varied among sites that showed moderate wilt infection, and included members in phylum *Gemmatimonadetes* (e.g., genus *Gemmatimonas*), *Firmicutes* (e.g., genus *Lysinibacillus*), *Acidobacteria* (e.g., genus *Gp16*), *Nitrospirae* (e.g., genus *Nitrospira*) and else. In soils with plants severely infected by bacterial wilt, the network maintaining taxa also varied.

**Table 2:** Important microbes in networks.

	Nodes with max degree	Nodes with max betweenness	Nodes with max stress centrality	Nodes with max eigenvector centrality
I	OTU_24040; Acidobacteria, Gp7	OTU_7856, Acidobacteria, Gp6	OTU_5231, Proteobacteria, Thiohalobacter	OTU_20616, Acidobacteria, Gp6
L	OTU_747, Chloroflexi, Thermomarinilinea	OTU_359, Acidobacteria, Gp25	OTU_747, Chloroflexi, Thermomarinilinea	OTU_340, Acidobacteria, Gp6
J	OTU_214, Acidobacteria, Gp4	OTU_909, Firmicutes, Bacillus	OTU_909, Firmicutes, Bacillus	OTU_214, Acidobacteria, Gp4
M	OTU_21670, Proteobacteria, Variovorax	OTU_113, Actinobacteria, Gaiella	OTU_113, Actinobacteria, Gaiella	OTU_478, Unclassified,
N	OTU_193, Proteobacteria, Hyphomicrobium	OTU_193 Proteobacteria, Hyphomicrobium	OTU_193, Proteobacteria, Hyphomicrobium	OTU_749, Proteobacteria, Azoarcus
C	OTU_24084, Proteobacteria, Curvibacter	OTU_720, Proteobacteria, Moraxellaceae(F)	OTU_720, Proteobacteria, Moraxellaceae(F)	OTU_214, Acidobacteria, Gp4
K	OTU_18948, Planctomycetes, Singulisphaera	OTU_18948, Planctomycetes, Singulisphaera	OTU_17, Firmicutes, Unclassified	OTU_21, Planctomycetes, Thermogutta
F	OTU_12483, Gemmatimonadetes, Gemmatimonas	OTU_204, Gemmatimonadetes, Gemmatimonas	OTU_204, Gemmatimonadetes, Gemmatimonas	OTU_43, Proteobacteria, Denitratisoma
G	OTU_123, Verrucomicrobia, Spartobacteria_genera_incertae_sedis OTU_36, Nitrospirae, Nitrospira	OTU_13176, Acidobacteria, Gp16	OTU_13176, Acidobacteria, Gp16	OTU_36, Nitrospirae, Nitrospira
H	OTU_229, Acidobacteria, Gp3	OTU_289, Firmicutes, Lysinibacillus	OTU_71, Chloroflexi, Unclassified	OTU_91, Bacteroidetes, Chitinophaga
D	OTU_2693, Proteobacteria, Ectothiorhodosinus	OTU_1565, Chloroflexi, Ktedonobacterales(O)	OTU_1565, Chloroflexi, Ktedonobacterales(O)	OTU_1399, Actinobacteria, Conexibacter
A	OTU_22415, Proteobacteria, Xanthomonadaceae (F)	OTU_5454 Armatimonadetes, Armatimonadetes_gp4	OTU_5454, Armatimonadetes, Armatimonadetes_gp4	OTU_4756, Chloroflexi, Ktedonobacter
B	OTU_183, Proteobacteria, Unclassified	OTU_24237, Planctomycetes, Rubinisphaera	OTU_24237, Planctomycetes, Rubinisphaera	OTU_82, Chloroflexi, Ktedonobacter
E	OTU_2035, Acidobacteria, Gp6	OTU_9618, Proteobacteria, Unclassified	OTU_9618, Proteobacteria, Unclassified	OTU_6, Verrucomicrobia, Spartobacteria_genera _incertae_sedis
O	OTU_45 Acidobacteria, Gp6	OTU_1301, Proteobacteria, Neisseriaceae(F)	OTU_1301, Proteobacteria, Neisseriaceae (F)	OTU_45, Acidobacteria, Gp6

*The classification levels are indicated in the brackets. O, oreder; F, family. If not specified, the taxa levels shown are phylum and genus.*

## Discussion

Multiple soil factors are responsible for bacterial wilt infection as the soil microbiome plays an important part of tobacco wilt severity. Because plants often lack genetic resistance to most necrotrophic pathogens, soil antagonistic microbes would protect the plants against pathogens (Cha et al., 2016). Pathogens of the genus *Ralstonia* are well-known for their ability to cause wilt symptoms in members of the *Solanaceous* family (Hayward, 1991; Chen et al., 2013). In this study, we focused on the relationship between the soil microbiome and tobacco *Ralstonia* wilt infection and found that wilt infection was closely related to the diversity, composition, and inferred microbe-microbe interactions of soil microbial communities.

Tobacco wilt infection was associated with soil microbial community diversity, as indicated by observed OTU number and Chao1 index. Bacterial OTUs were more diverse in fields with healthy plants than in fields with infection. Even though the correlation between the Shannon index and wilt infection was not strong, our results showed that the microbial community diversity had a positive correlation with wilt infection rate. This is similar to a recent report that the microbial community diversity was higher in soils with healthy plants than in soils with plants infected by bacterial wilt (Wang et al., 2017). It has also been reported that the rhizosphere soil microbial community diversity is positively correlated with plant health (Bulluck and Ristaino, 2002; Luan et al., 2015). The results suggest that a highly diverse soil microbiome (greater numbers of microbial species) would decrease the chances of bacterial wilt outbreak. Many studies have also found that a diverse microbial community was often less prone to pathogens invasion than a simpler microbial community (Case, 1990; Fargione and Tilman, 2005; van Elsas et al., 2012).

Interestingly, all filed sites clustered into ‘healthy’ and ‘infected groups, in the UPGMA tree or the first PCA axis and that this clustering was not related to either the geographic locations or cultivation regimes. The ‘healthy’ (0–25%) branch of soil samples was from Huahenger (C), Pailiao (I and J) or Fenghuang (L, M, and N), and ‘infected’ branch (>25%) was based on rotations (D and E) or continuous (A and B) cultivation regimes. These results suggested that soil microbial community composition could have played a role in preventing tobacco from wilt pathogens infections. This may be because certain populations of beneficial soil microbes were enhanced in sites with low incidence of bacterial wilt. For example, Bulluck and Ristaino (2002) found that the increase of beneficial soil microbes significantly reduced the outbreak chance of southern blight. In this context, soil microbial communities with abundant biocontrol agents (BCAs) or pathogenic antagonists may be able to control the incidence of soil borne bacterial diseases. Even though not all BCAs or pathogenic antagonists are known and some BCAs or pathogenic antagonistic members could become plant pathogens under specific circumstances, we can propose that soils with a similar microbial community structure of the ‘healthy’ branch are likely to suppress the outbreak of tobacco wilt disease.

To further reveal which soil microbes may be important for inhibiting wilt disease outbreaks, we attempted to identify ‘inferred’ key microbial groups in molecular ecological networks. In networks, nodes with max connectivity (degree), max betweenness, stress centrality and eigenvector centrality play important roles in maintaining network structure (Olesen et al., 2007; Zhou et al., 2011; Faust and Raes, 2012). This is because (i) nodes with max degree have the most edges in the network, (ii) nodes with max betweenness and stress centrality can serve as brokers in the network and (iii) nodes with max eigenvector centrality have the highest connections with other central nodes. Furthermore, it is also supposed that the taxa of these nodes play important roles in maintaining ecosystem stability (Olesen et al., 2007; Lu et al., 2013). In this study, these key taxa were relatively consistent in fields with healthy plants (branch 1 on the UPGMA tree), suggesting that these taxa might be important in the suppression of bacteria wilt disease. In fields with healthy plants, taxa considered important for maintaining ecosystem stability were mainly *Bacillus* in the phylum *Firmicutes*, several members in the phylum *Actinobacteria* and a few other organisms. These taxa are often considered as plant-beneficial microbes. Members of the genus *Bacillus* are well-known as BCAs (Emmert and Handelsman, 1999; Schisler et al., 2004; Ongena and Jacques, 2008). Due to their ability to secrete antibiotics or antimicrobial proteins (Ahimou et al., 2000; Moyne et al., 2004), they showed a wide range of biological effects on plant pathogens and have been applied to control bacterial diseases of alfalfa (Handelsman et al., 1990), tobacco (Fravel et al., 1977) and cucumber (Cao et al., 2011). Many members of *Actinobacteria* have also shown the ability to control plant bacterial diseases (Gomes et al., 2000; Doumbou et al., 2001; Kinkel et al., 2012; Palaniyandi et al., 2013). *Actinobacteria* are well-known antagonistic microbes of many pathogens, as they can (i) produce a diverse range of antibiotics (Liu et al., 2012), (ii) secret cell-wall degrading enzymes (Chater et al., 2010), and/or (iii) induce host (plant) resistance (Conn et al., 2008). In the wilt disease infected fields (branch 2), key taxa for maintaining ecosystem stability varied. As sites with higher wilt infection rate formed simple networks (fewer edges and lower average degree) with fewer modules, it is possible that some key bacteria in disease-causing soils inhibited the formation of complex, modular networks that were characteristic of suppressive soils. In this context, we proposed that they could possibly promote the formation of complex, modular microbial community networks, thus making soils wilt-suppressive when plant-beneficial taxa played important roles in maintaining the ecosystem stability. Taking the microbial community composition and the important taxa together, the results indicate that when the soil microbial community composition is similar to that of the ‘healthy’ group with plant-beneficial microbes, the soil ecosystem may remain stable and disease-suppressive.

We proposed that complex, modular microbial community networks might make soils wilt-suppressive, however, since the soil ecology is very complex, with the flow of energy, matter, and information not well understood a majority of the soil microbes still unculturable, most of the microbe–microbe interactions remain unclear. More research is still needed to reveal the relationship between below-ground ecology networks and above-ground plant health. Previous studies have indicated that networks could be altered by environmental properties, such as pH (Tylianakis et al., 2007; Barberán et al., 2012; Shi et al., 2016). This was consistent with the present study which showed a significant correlation between soil pH and network complexity (Supplementary Table S5). We also observed that the bacterial community diversity showed significant positive correlation with the network complexity, which is, however, in contrast with the finding in rhizosphere networks (Shi et al., 2016) whose complexity was accompanied by decreased bacterial diversity. This might be because the rhizosphere and the surrounding bulk soils have very different bacterial communities (Kourtev et al., 2002; Berg and Smalla, 2009; Shi et al., 2015) and ecological networks were more complicated in rhizosphere than in surrounding bulk soils.

**FIGURE 5:**
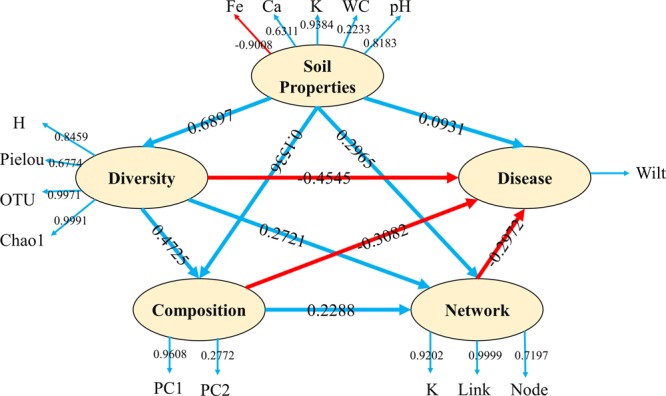
Partial Least Squares Path Modeling (PLSPM). Blue lines represent positive effects and red lines represent negative effect. Numbers on the lines in the PLSPM model are the ‘total effects’ values. Numbers on the lines out of the PLSPM model are the ‘weight’ contributions. WC, water content; H, Shannon diversity index; K, average degree.

The present study showed a range of correlations between plant wilt infection and soil microbial properties. We conducted PLSPM analyses (**Figure 5**) to profile the complex relationship between soil microbial community properties and plant health. Soil properties had strong effect on microbial diversity and weak direct effect on microbial composition, microbial networks and disease infections, however, soil properties can also affect plant disease infection indirectly through soil microbial diversity, composition and network interactions. Among soil microbial properties, microbe diversity showed the strongest effects (negatively, -0.4545) on plant disease infection, followed by microbial community composition (negatively, -0.3082 and molecular ecology network complexity (negatively, -0.2972).

In summary, the present study offered an integrate view of the relationship between soil bacterial community and plant health. We found that (i) soil microbial diversity had a strong effect on plant disease level, with diversity and rate of wilt disease showing an inverse relationship; and (ii) soils with similar microbial community composition have similar disease infection rate. When the soil had similar composition with ‘healthy’ group, the soil might tend to be disease-suppressive. According to the inferred molecular ecology networks, we proposed that a more complex network might be beneficial for tobacco wilt suppression. In addition, some potential plant-beneficial microbial groups could act as network key taxa, thus reducing the chance of plant soil-borne pathogen invasion. However, as the soil microbial ecology network are extremely complex, more work, particularly the experimental work, is still needed to test the proposal. We concluded that microbial community in disease-suppressive soils may be consisted with high diversity, consistent composition with plant-beneficial microbes as the important component and complex network.

## Author Contributions

HYa, YL, HL, and HYi conceived and designed the experiments. YL, HL, XL, and HYi contributed reagents and materials. JH, JL, and YG performed the experiments. DM, YG, and YX analyzed the data. DM, JH, YX, and HYi wrote and revised the paper.

## Conflict of Interest Statement

The authors declare that the research was conducted in the absence of any commercial or financial relationships that could be construed as a potential conflict of interest.

## Footnotes

1 http://www.ou.edu/content/ieg/tools/data-analysis-pipeline.html

2 http://129.15.40.240/MENA/
