# Supplementary material for: An Integrated Insight into the Relationship between Soil Microbial Community and Tobacco Bacterial Wilt Disease
Source: Front Microbiol. 2017 Nov 7;8:2179. doi: 10.3389/fmicb.2017.02179 (PMC5681905; doi:10.3389/fmicb.2017.02179)
Supplement: Supplementary file 1 [file Data_Sheet_1.DOCX]

Supporting information for

An integrated insight into the relationship between soil microbial community and tobacco bacterial wilt disease

Hongwu Yang^1a^, Juan Li^1a^, Yunhua Xiao^2,3^, Yabing Gu^2,3^, Hongwei Liu^2,3^, Yili Liang^2,3^, Xueduan Liu^2,3^, Jin Hu^2,3^, Delong Meng^2,3^*, Huaqun Yin^2,3^*,

^1^College of Agronomy, Hunan Agricultural University, Changsha, China

^2^School of Minerals Processing and Bioengineering, Central South University, Changsha, China

^3^Key laboratory of Biometallurgy, Ministry of Education, Changsha, China

Running title: Soil microbial community and plant health

^a^Authors contribute equally to present work.

*Corresponding Author:

Huaqun Yin

Email: [yinhuaqun_cs@sina.com](mailto:yinhuaqun_cs@sina.com)

Delong Meng

Tel: +86(731)88830546; Fax: +86(731)88830546. E-mail: delong.meng@gmail.com





Figure S1 Correlation between soil properties (pH and Ca) and wilt disease.


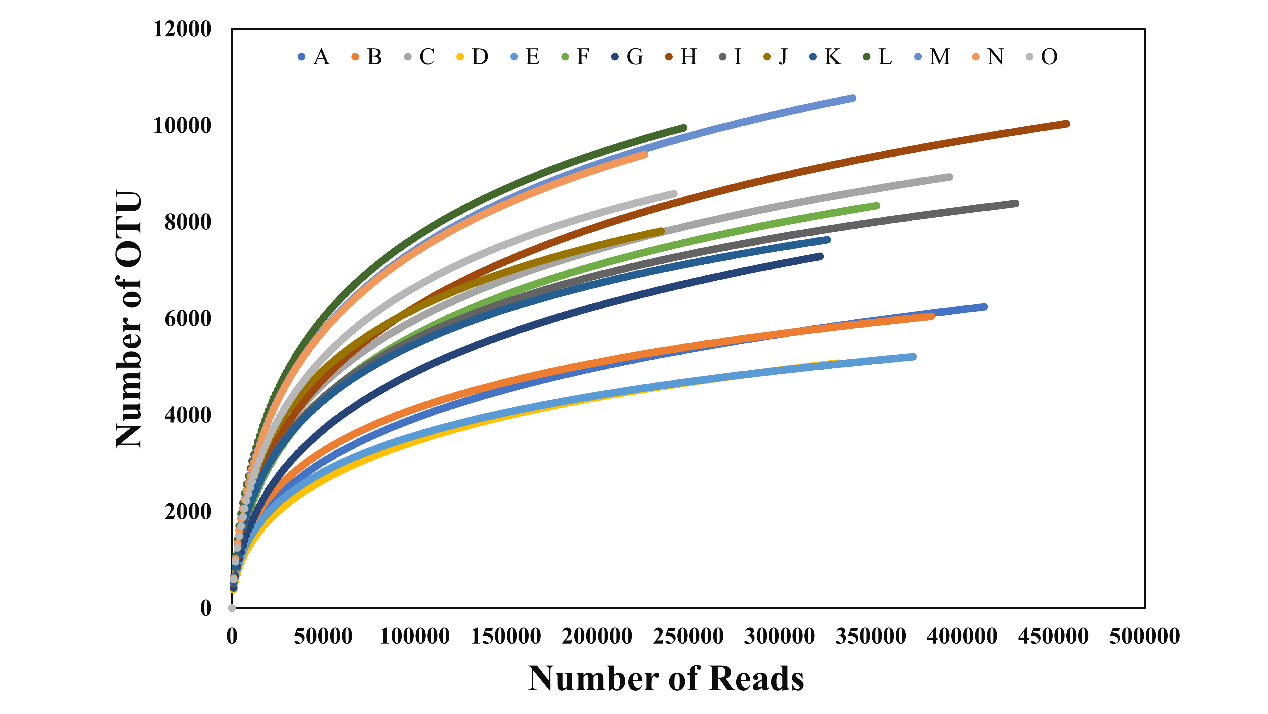


Figure S2 Rarefaction curve.

Table S1 Description of sampling 15 sampling fields.

| Sample ID | Wilt infection, % | Sample Location | No. of sample Cores | Longitude | Latitude | Altitude | Cultivation type | Soil pH | Soil physics |
| --- | --- | --- | --- | --- | --- | --- | --- | --- | --- |
| A | 57.78 | Huahenger | 10 | 109°27′5″ | 28°30′57″ | 497 | Continues | 5.06 | Sandy |
| B | 57.78 | Huahenger | 10 | 109°27′5″ | 28°30′57″ | 497 | Continues | 5.40 | Sandy |
| C | 23.54 | Huahenger | 10 | 109°27′13″ | 28°31′3″ | 494 | Rotation | 4.79 | Clay |
| D | 48.67 | Huahenger | 9 | 109°27′13″ | 28°31′5″ | 495 | Rotation | 4.46 | Clay |
| E | 59.62 | Huahenger | 10 | 109°27′11″ | 28°31′10″ | 492 | Rotation | 4.62 | Clay |
| F | 40.09 | Pailiao | 10 | 109°34′36″ | 28°22′11″ | 766 | Continues | 5.15 | Loam |
| G | 40.73 | Pailiao | 10 | 109°34′36″ | 28°22′11″ | 766 | Continues | 5.16 | Loam |
| H | 34.48 | Pailiao | 10 | 109°34′36″ | 28°22′11″ | 765 | Continues | 5.17 | Loam |
| I | 1.19 | Pailiao | 10 | 109°34′41″ | 28°22′12″ | 765 | Continues | 6.12 | Loam |
| J | 7.26 | Pailiao | 8 | 109°34′41″ | 28°22′12″ | 768 | Continues | 6.15 | Loam |
| K | 39.98 | Pailiao | 10 | 109°34′13″ | 28°22′8″ | 799 | Continues | 5.54 | Loam |
| L | 3.38 | Fenghuang | 10 | 109°29′59″ | 28°2′0″ | 479 | Rotation | 5.35 | Loam |
| M | 8.78 | Fenghuang | 10 | 109°29′59″ | 28°2′0″ | 479 | Rotation | 5.45 | Loam |
| N | 22.97 | Fenghuang | 10 | 109°29′59″ | 28°2′0″ | 479 | Rotation | 5.76 | Loam |
| O | 80.45 | Fenghuang | 10 | 109°30′11″ | 28°2′05″ | 479 | Continues | 5.10 | Loam |

Table S2 Soil properties in 15 sampling sites.

| Site | Wilt infection, % | Water Content, % | PH | K, mg Kg^-1^ | Ca, mg Kg^-1^ | Fe, mg Kg^-1^ |
| --- | --- | --- | --- | --- | --- | --- |
| A | 57.78 | 15.74 | 5.055 | 14135 | 1102 | 26391 |
| B | 57.78 | 15.52 | 5.396 | 11882 | 977 | 20297 |
| C | 23.54 | 15.99 | 4.787 | 14300 | 1552 | 28582 |
| D | 48.67 | 15.97 | 4.463 | 14541 | 1033 | 31240 |
| E | 59.62 | 15.86 | 4.624 | 14823 | 1130 | 32353 |
| F | 40.09 | 16.46 | 5.151 | 23969 | 2036 | 15465 |
| G | 40.73 | 16.51 | 5.159 | 26414 | 2013 | 16901 |
| H | 34.48 | 20.72 | 5.1675 | 26081 | 1618 | 17071 |
| I | 1.19 | 15.71 | 6.117 | 27489 | 10199 | 18680 |
| J | 7.26 | 17.13 | 6.15 | 26271 | 15146 | 14824 |
| K | 39.98 | 19.57 | 5.544 | 24542 | 1203 | 14776 |
| L | 3.38 | 14.42 | 5.346 | 24758 | 2770 | 15660 |
| M | 8.78 | 14.25 | 5.451 | 24003 | 2653 | 15475 |
| N | 22.97 | 15.62 | 5.763 | 24058 | 2474 | 15806 |
| O | 80.45 | 17.07 | 5.09875 | 23980 | 1581 | 15277 |

Table S3 Pearson correlation between wilt infection rate and phylum

| Phylum | Pearson | p |  | Phylum | Pearson | p |
| --- | --- | --- | --- | --- | --- | --- |
| Acidobacteria | -0.629 | 0.012 |  | Fusobacteria | 0.426 | 0.113 |
| Actinobacteria | 0.697 | 0.004 |  | Gemmatimonadetes | -0.011 | 0.969 |
| Aminicenantes | -0.421 | 0.118 |  | Hydrogenedentes | -0.559 | 0.030 |
| Aquificae | -0.433 | 0.107 |  | Ignavibacteriae | -0.607 | 0.016 |
| Armatimonadetes | -0.296 | 0.284 |  | Latescibacteria | -0.651 | 0.009 |
| Bacteroidetes | -0.163 | 0.561 |  | Microgenomates | -0.450 | 0.092 |
| BRC1 | -0.762 | 0.001 |  | Nitrospirae | -0.224 | 0.423 |
| candidate division WPS-1 | 0.333 | 0.263 |  | Omnitrophica | -0.509 | 0.053 |
| candidate division WPS-2 | 0.439 | 0.038 |  | Pacearchaeota | -0.737 | 0.002 |
| Candidatus Saccharibacteria | 0.398 | 0.173 |  | Parcubacteria | 0.108 | 0.700 |
| Chlamydiae | 0.371 | 0.174 |  | Planctomycetes | -0.172 | 0.539 |
| Chloroflexi | -0.179 | 0.523 |  | Proteobacteria | 0.358 | 0.190 |
| Crenarchaeota | -0.573 | 0.026 |  | Spirochaetes | 0.146 | 0.602 |
| Cyanobacteria | 0.297 | 0.282 |  | SR1 | 0.148 | 0.598 |
| Deferribacteres | 0.231 | 0.408 |  | Synergistetes | -0.677 | 0.006 |
| Deinococcus-Ther | -0.383 | 0.158 |  | Tenericutes | -0.09 | 0.750 |
| Elusimicrobia | -0.302 | 0.274 |  | Thaumarchaeota | -0.424 | 0.116 |
| Euryarchaeota | -0.612 | 0.015 |  | Thermodesulfobac | 0.381 | 0.161 |
| Fibrobacteres | -0.175 | 0.533 |  | Verrucomicrobia | 0.140 | 0.618 |
| Firmicutes | 0.411 | 0.129 |  | Woesearchaeota | -0.546 | 0.035 |

Table S4 Network properties. Mor: morbidity; St similarity threshold; Nodes: number of nodes; Links: number of links; avgK: average degree; avgCC: average clustering coefficient; GD: average path distance; E: Geodesic efficiency; HD: Harmonic geodesic distance; CD: Centralization of degree; CB: Centralization of betweenness; CS centralization of stress centrality; Max.E: Maximal eigenvector centrality; CE: Centralization of eigenvector centrality; D: Density; Trans: Transitivity; Con: Connectedness; E: Efficiency.

|  | I | L | J | M | N | C | H | K | F | G | D | A | B | E | O |
| --- | --- | --- | --- | --- | --- | --- | --- | --- | --- | --- | --- | --- | --- | --- | --- |
| Mor | 1.19 | 3.38 | 7.26 | 8.78 | 22.97 | 23.54 | 34.48 | 39.98 | 40.09 | 40.73 | 48.67 | 57.78 | 57.78 | 59.62 | 80.45 |
| St | 0.93 | 0.94 | 0.96 | 0.94 | 0.94 | 0.92 | 0.92 | 0.93 | 0.94 | 0.89 | 0.92 | 0.92 | 0.93 | 0.93 | 0.93 |
| Modules | 122 | 55 | 187 | 208 | 30 | 127 | 62 | 91 | 67 | 97 | 56 | 80 | 55 | 82 | 47 |
| Nodes | 1182 | 985 | 1436 | 652 | 398 | 956 | 674 | 718 | 758 | 794 | 582 | 833 | 749 | 602 | 1040 |
| Links | 3550 | 5480 | 12850 | 2850 | 1559 | 1536 | 1417 | 2319 | 1490 | 1586 | 1139 | 1813 | 2114 | 1146 | 1220 |
| R^2^ | 0.92 | 0.82 | 0.87 | 0.85 | 0.86 | 0.91 | 0.93 | 0.87 | 0.92 | 0.93 | 0.90 | 0.91 | 0.89 | 0.91 | 0.93 |
| avgK | 6.01 | 11.13 | 17.90 | 8.74 | 7.83 | 3.21 | 4.21 | 6.46 | 3.93 | 4.00 | 3.91 | 4.35 | 5.65 | 3.81 | 2.35 |
| avgCC | 0.27 | 0.37 | 0.25 | 0.34 | 0.29 | 0.17 | 0.24 | 0.25 | 0.24 | 0.20 | 0.24 | 0.24 | 0.27 | 0.21 | 0.12 |
| GD | 8.4 | 3.8 | 4.8 | 5.3 | 4.2 | 10.6 | 7.0 | 4.9 | 8.9 | 7.6 | 5.8 | 8.5 | 6.7 | 10.2 | 9.4 |
| E | 0.18 | 0.33 | 0.31 | 0.26 | 0.30 | 0.13 | 0.19 | 0.26 | 0.16 | 0.19 | 0.24 | 0.15 | 0.20 | 0.17 | 0.15 |
| HD | 5.48 | 3.07 | 3.27 | 3.85 | 3.34 | 7.51 | 5.23 | 3.80 | 6.27 | 5.41 | 4.20 | 6.49 | 5.13 | 5.98 | 6.90 |
| CD | 0.06 | 0.21 | 0.17 | 0.22 | 0.19 | 0.03 | 0.05 | 0.14 | 0.09 | 0.04 | 0.06 | 0.03 | 0.09 | 0.10 | 0.03 |
| CB | 0.11 | 0.07 | 0.04 | 0.16 | 0.22 | 0.19 | 0.11 | 0.08 | 0.20 | 0.13 | 0.04 | 0.18 | 0.12 | 0.24 | 0.05 |
| CS | 41.568 | 8.84 | 3.239 | 8.985 | 5.032 | 7.009 | 5.085 | 3.121 | 3.835 | 0.959 | 0.269 | 9.161 | 5.788 | 6.839 | 0.583 |
| Max.E | 0.21 | 0.20 | 0.17 | 0.24 | 0.23 | 0.29 | 0.28 | 0.26 | 0.34 | 0.28 | 0.33 | 0.26 | 0.30 | 0.33 | 0.30 |
| CE | 0.20 | 0.19 | 0.15 | 0.23 | 0.20 | 0.28 | 0.27 | 0.24 | 0.33 | 0.27 | 0.32 | 0.25 | 0.29 | 0.31 | 0.29 |
| D | 0.005 | 0.011 | 0.012 | 0.013 | 0.02 | 0.003 | 0.006 | 0.009 | 0.005 | 0.005 | 0.007 | 0.005 | 0.008 | 0.006 | 0.002 |
| Trans | 0.36 | 0.14 | 0.29 | 0.22 | 0.38 | 0.31 | 0.30 | 0.30 | 0.22 | 0.28 | 0.23 | 0.38 | 0.26 | 0.25 | 0.26 |
| Con | 0.41 | 0.70 | 0.37 | 0.72 | 0.70 | 0.46 | 0.56 | 0.51 | 0.60 | 0.51 | 0.29 | 0.64 | 0.70 | 0.51 | 0.23 |
| E | 0.99 | 0.99 | 0.97 | 0.98 | 0.98 | 1.00 | 0.99 | 0.99 | 0.99 | 0.99 | 0.98 | 0.99 | 0.99 | 0.99 | 0.99 |

Table S5 Pearson correlation matrix of sizes, which are related to soil properties (pH), plant health (Wilt) and soil microbial communities (alpha-diversity, beta-diversity and network properties). Average K: average degree.

| Pearson correlation matrix | | pH | Wilt | Alpha-Diversity | | | | Beta-Diversity | | Network | |
| --- | --- | --- | --- | --- | --- | --- | --- | --- | --- | --- | --- |
|  |  |  |  | Diversity | Evenness | Observed OTU | Chao1 | PC1 | PC2 | Nodes | Links |
| Morbidity | Pearson | -0.606 |  |  |  |  |  |  |  |  |  |
|  | p value | 0.017 |  |  |  |  |  |  |  |  |  |
| Diversity | Pearson | 0.532 | -0.485 |  |  |  |  |  |  |  |  |
|  | p value | 0.041 | 0.067 |  |  |  |  |  |  |  |  |
| Evenness | Pearson | 0.497 | -0.402 | 0.977 |  |  |  |  |  |  |  |
|  | p value | 0.059 | 0.137 | 0 |  |  |  |  |  |  |  |
| Observed  OTU | Pearson | 0.623 | -0.751 | 0.787 | 0.690 |  |  |  |  |  |  |
|  | p value | 0.013 | 0.001 | 0 | 0.004 |  |  |  |  |  |  |
| Chao1 | Pearson | 0.600 | -0.747 | 0.766 | 0.654 | 0.993 |  |  |  |  |  |
|  | p value | 0.018 | 0.001 | 0.001 | 0.008 | 0 |  |  |  |  |  |
| PC1 | Pearson | 0.682 | -0.639 | 0.58 | 0.584 | 0.638 | 0.567 |  |  |  |  |
|  | p value | 0.005 | 0.01 | 0.023 | 0.022 | 0.011 | 0.027 |  |  |  |  |
| PC2 | Pearson | 0.223 | -0.252 | -0.417 | -0.516 | -0.009 | 0.051 | 0 |  |  |  |
|  | p value | 0.425 | 0.365 | 0.122 | 0.049 | 0.974 | 0.858 | 1 |  |  |  |
| Nodes | Pearson | 0.473 | -0.317 | 0.215 | 0.211 | 0.470 | 0.429 | 0.473 | 0.239 |  |  |
|  | p value | 0.075 | 0.250 | 0.442 | 0.451 | 0.077 | 0.111 | 0.075 | 0.390 |  |  |
| Links | Pearson | 0.667 | -0.684 | 0.511 | 0.526 | 0.631 | 0.595 | 0.544 | 0.010 | 0.711 |  |
|  | p value | 0.007 | 0.005 | 0.051 | 0.044 | 0.012 | 0.019 | 0.036 | 0.971 | 0.003 |  |
| Average K | Pearson | 0.680 | -0.656 | 0.566 | 0.600 | 0.58 | 0.549 | 0.469 | -0.102 | 0.488 | 0.923 |
|  | p value | 0.005 | 0.008 | 0.028 | 0.018 | 0.023 | 0.034 | 0.078 | 0.719 | 0.065 | 0 |
